# Dysregulation of Endothelin-1: Implications for Health Disparities in Alzheimer’s Disease

**DOI:** 10.3390/jpm10040199

**Published:** 2020-10-28

**Authors:** Donald J. Alcendor

**Affiliations:** Center for AIDS Health Disparities Research, Department of Microbiology, Immunology and Physiology, School of Medicine, Meharry Medical College, Nashville, TN 37208, USA; dalcendor@mmc.edu

**Keywords:** Endthelin-1, Alzheimer’s disease, infection, mortality, minorities, health disparities, health inequities, African Americans, Hispanics/Latinos, non-Hispanic Whites

## Abstract

Alzheimer’s disease (AD) and related dementias disproportionately impact racial and ethnic minorities. The racial and ethnic disparities in AD could be explained by differences in cerebral vascular disease pathology. Endothelin-1 (ET-1) is a potent vasoconstrictive peptide that regulates smooth muscle, endothelial cell, and pericyte contractions that may result in cerebral vascular constriction, leading to cerebral hypoperfusion; over time, ET-1 may result in neuronal injury contributing to the pathology of AD. Upregulation of the ET-1 system has been observed in African Americans when compared with non-Hispanic Whites. The role of the ET-1 system as a driver of ethnic disparities in AD requires further investigation. Targeting of the ET-1 system as a therapeutic intervention that could impact AD progression also needs further study. Dysregulation of ET-1 in Hispanic/Latino populations largely have been unexplored. Genetics linking ET-1 dysregulation and racial disparities in AD also needs further investigation. In this review, I examine how AD effects underserved minority populations and how dysregulation of the ET-1 system specifically predisposes ethnic minorities to AD. In addition, I examine the molecular interactions of the ET-1 system and amyloid beta, the role the ET-1 system in neurodegeneration, potential therapeutics for ET-1 dysregulation, and the impact on AD progression.

## 1. Background/Introduction

Alzheimer’s disease (AD) and related dementias (ADRD) disproportionately impact racial and ethnic minority and socioeconomically disadvantaged adults [1,2,3,4,5]. Racial and ethnic disparities in AD could be explained by differences in vascular disease pathology. Upregulation of the endothelin system is present in African Americans (AAs) when compared with non-Hispanic Whites (NHWs) [6]. Endothelin-1 (ET-1) is a potent vasoconstrictive peptide that is released basolaterally primarily by endothelial cells to induce vascular smooth muscle cells and pericyte contractions [7,8,9]. Pericytes expressed BQ-123-sensitive ET_A_ receptors for endothelins, as evidenced by ^125^I-Et-1 binding experiments [10]. Dysregulation of the endothelin system occurs in AD. However, AAs experience higher vasoconstriction-mediated blood pressure in response to acute stress than NHWs. A study by Treiber et al. that examined hemodynamic and plasma ET-1 levels in AA and NHW males revealed that AAs had higher resting plasma ET-1 levels and a greater increase in ET-1 in response to stressors [6]. The data supports the notion that racial differences exist in hemodynamic reactivity to stress between AAs and NHWs [6]. AA adults with hypertension have been shown to have significantly higher ET-1 levels than normotensive AAs and NHWs [6]. Ergul et al. discovered that AAs have a higher ratio of vasoconstriction-promoting ET receptors in saphenous veins when compared with NHWs, regardless of whether they were hypertensive, which implicates racial differences in peripheral vascular resistance and diminished vasodilatation in response to environmental stress [11]. These findings suggest that AAs are at higher risk for cardiovascular disease than NHWs, which also increases their risk of dementia. Diabetes mellitus (DM) is associated with an increased risk of cognitive decline and AD later in life. Studies have shown that type 2 DM (T2DM) nearly doubles the risk of AD, and it is well known that a health disparity exists among AAs for T2DM when compared with NHWs [12]. Signal peptide-CUB-EGF-like-containing protein 2 (SCUBE2) has been shown to be upregulated in atherosclerotic human coronary artery disease [13]. Ali et al. examined SCUBE2 expression in T2DM patients with dyslipidemia and found that upregulation of SCUBE2 expression in dyslipidemic T2DM is associated with higher levels of ET-1 when compared with controls [13]. Kostov et al. examined serum concentrations of ET-1 and matrix metalloproteinases-2, -9 in pre-hypertensive and hypertensive patients with T2DM [14]. They demonstrated increased levels of ET-1 in patients with T2DM when compared with controls, suggesting that high levels of ET-1 could lead to long-lasting increases in blood pressure and clinical manifestation of hypertension [14]. Palmer et al. revealed that ET-1 levels are elevated in AD and are upregulated by amyloid β (Aβ); they also demonstrated that endothelin-converting enzyme-2 (ECE-2) is upregulated by Aβ, and its expression is elevated in AD post-mortem brain tissue when compared with controls [15]. Furthermore, ET-1 expression is induced by hypoxia or ischemia caused by pericyte-mediated constriction of brain vascular capillaries [16]. Here, I examine the role of the ET-1 system in the development of neuronal injury that contributes to the pathology of AD and possible ADRDs.

## 2. Underserved Minority Populations and AD

Regardless of the health disparities associated with AD among ethnic minorities, large population-based studies that examined the pathobiology of AD have been performed with predominantly NHWs, resulting in extrapolation of research findings to minority populations [17,18]. Aging and genetics are important risk factors; however, other demographic factors in the United States (U.S.) are rising for AD, including a surge in Hispanic/Latino (H/L) populations [19,20]. Several studies reveal a higher prevalence of AD in AAs and H/Ls [21,22,23]. Approximately 12% of older adults in the H/L population are diagnosed with AD, which is the highest among all ethnic groups in the U.S. and represents an H/L health disparity [24,25]. Despite genetic and cultural diversity among H/L populations, they display an earlier age of onset when compared with NHWs [26,27]. Vega et al. examined the interplay between social determinants of health, co-morbidities, and genetic factors associated with AD in H/L communities [28]. Understanding the role of these factors could help to explain this populations’ increased risk. While the burden of U.S. disparities in AD is associated directly with social disparities, racial and ethnic disparities could be explained in part by differences in susceptibility to vascular disease.

## 3. ET-1 System and Regulation

**Figure 1:**
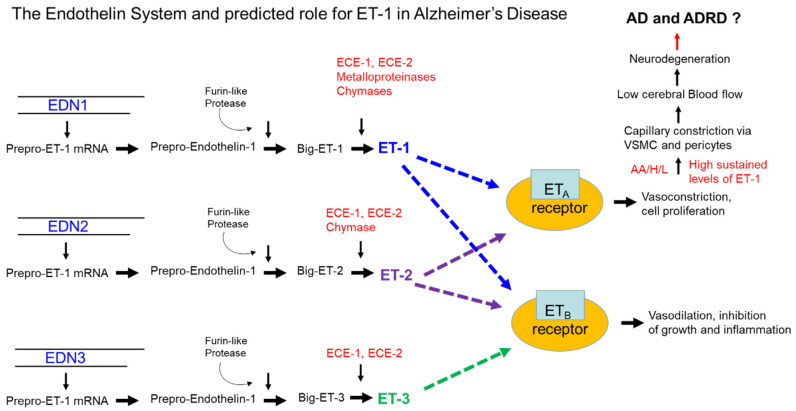
The endothelin system and its potential role in Alzheimer’s disease (AD). The synthesis of Endothelin-1 (ET-1), ET-2, and ET-3 occurs from separate mRNAs encoded by *EDN-1*, *EDN-2*, and *END-3*. These preproendothelin mRNAs 1–3 are translated to form preproendothelins 1–3. The three proteins are cleaved via furin-like protease to form Big ETs (Big ET-1, Big ET-2, and Big ET-3). Big-ETs are converted to the active peptide forms of ET-1 via ECE-1 and ECE-2, as well as by metalloproteinases and chymases, ET-2 via ECE-1, ECE-2 and chymase, and ET-3 via ECE-1 and ECE-2, respectively. The active forms bind to cognate G-protein receptors ET_A_ and ET_B_ to activate cellular functions via ET_B_ receptor binding by ET-1–3, resulting in vasodilation and inhibition of growth and inflammation; ET-1 and ET-2 may activate cellular function by binding to ET_A_ receptors on vascular smooth muscle cells (VSMC) and pericytes in the brain and induce vasoconstriction, and cell proliferative responses. High sustained levels of ET-1 among AA and possibly Hispanic/Latino (H/L) populations at high risk for AD could predispose them to conditions of low cerebral blood flow (CBF), leading to neurodegeneration that could contribute to AD and Alzheimer’s disease related dementias (ADRD).

**Figure 2:**
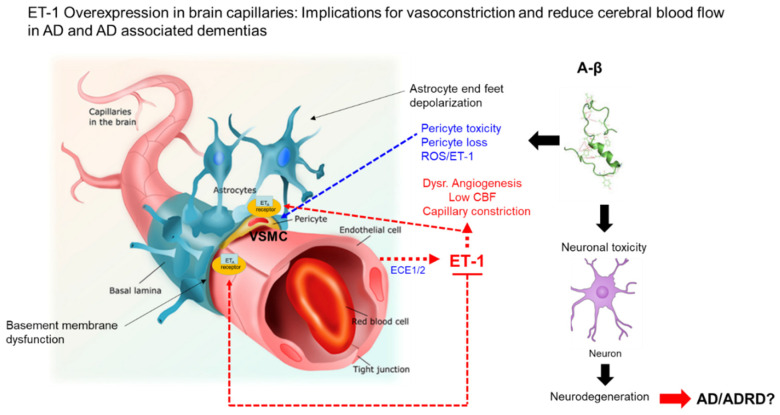
Pathophysiology in dysregulation of the ET-1 system with implications for AD. A theoretical model of the effects of ET-1 and Aβ proteins on VSMC and pericyte functions, respectively, in capillaries and components of the neurovascular unit. The effects of ET-1 on VSMC and pericytes include vasoconstriction, the disruption of angiogenesis leading to low CBF. The effects of Aβ proteins on pericytes include pericyte loss and toxicity, induction of ROS, and capillary constriction, all contributing to neuronal toxicity and neurodegeneration that may contribute to AD and ADRD. Note: Figure 2, was revised from a previous report by Alcendor [53]. For the original figure, I acknowledge Pearson Education, Inc. (2014).

ET-1 was first isolated by Yanagisawa et al. in 1988 from cell culture supernatant of porcine aortic endothelial cells; the team referred to the factor as endothelin, which is now referred to as ET-1 [29]. Yanagisawa et al. determined that a larger peptide of 39 amino acids represented a precursor of the smaller mature bioactive of ET-1 (29 amino acids), named Big Endothelin (Big ET-1) [29] (Figure 1). Big ET-1 was found to undergo proteolytic cleavage by ECE-1. ET-1 was recognized as a potent, long-lasting endothelium-derived vasoconstrictive peptide and is the principal isoform in the human cardiovascular system [29]. Via two G protein-coupled receptors, endothelin receptor type A (ET_A_) and endothelin receptor type B (ET_B_), ET-1 may induce vasoconstriction, vasodilation, and vascular cell proliferation on resident vessels of the vascular system [30,31,32,33]. Along with its effects on vascular endothelial cells, ET-1 also may induce vascular smooth cell proliferation, mediated via ET_A_ and ET_B_ [34,35] (Figure 1). As described, the ET system consists of three major components: (1) ET-1; (2) ECE-1 (four isoforms of ECE-1 exist in humans (ECE-1a, ECE-1b, ECE-1c, and ECE-1d)), which is responsible for the biosynthesis of the active ET peptide [36]; and (3) ET receptors (ET_A_ and ET_B_), which mediate the biological effects of this peptide [37,38] (Figure 1). In disease states, dysregulation of receptor expression may lead to uncontrolled vasoconstriction and cell proliferation by ET-1. The endothelin family is comprised of three members (ET-1, ET-2, and ET-3) of structurally similar 21 amino acid peptides that are cleaved from their respective Big ET-1 precursors [39]. ET-1 and ET-2 both bind ET_A_ and ET_B_ receptors with strong affinity [40], but ET-3 has a lower affinity for the ET_B_ receptor [41] (Figure 1). The focus in this review is on ET-1, which is the parent compound of the endothelin family. ET-1 is not stored in cellular compartments but is generated in response to stimuli that include, but are not limited to, vasoconstrictive mediators, thrombogenic agents, cytokines, and growth factors, physiochemical factors, hormones, and certain drugs [42]. ET-1 is constitutive, secreted by endothelial cells, and released abluminally (Figure 2). ET-1 is regulated at the levels of transcription and has a half-life of approximately 15–20 min; however, its biological effects may last for two hours due to its affinity for its receptor post-endocytosis [42]. Venous plasma levels of ET-1 may be useful as an index for endothelial synthesis [42]. ET-1 clearance occurs by pulmonary and renal vascular beds via the ET_B_ receptors through internalization and lysosomal degradation. Along with other endothelins, ET-1 also is degraded by neutral endopeptidases, which are mainly found in the brush border vesicles of kidney proximal tubules. This is significant in that individuals with chronic kidney disease have elevated plasma ET-1 levels; however, little or no changes in Big ET-1 levels were observed. ET-1 has two intramolecular disulfide bonds between cysteine residues that are crosslinked between positions 1 and 15 and 3 and 11, which is distinct among vasoactive mammalian peptides [43,44,45]. The binding of ET-1 to ET_A_ and ET_B_ receptors requires prior proteolytic cleavage by ECE-1 from Big ET-1 to facilitate receptor binding [46] (Figure 2). The abnormal folding of Big ET-1 prevents receptor interaction, and unlike the mature ET-1, Big ET-1 is resistant to proteolytic cleavage [47]. The different ET isoforms (ET-1, ET-2, and ET-3) are synthesized in three steps. Translation of a prepropeptide is present that undergoes proteolytic cleavage to yield a propeptides that are further cleaved by the enzyme furin to yield the Big ET precursors [48] (Figure 1). For example, Big ET-1 is cleaved by ECE-1 to yield the mature, biologically active ET-1. Notably, Big ET-1 is cleaved by ECE-1 at pH 7 and also by ECE-2 at pH 5.5 in endothelial cells [49]. Davenport et al. report that after release of ET-1 from endothelial cells, one in five molecules of Big ET-1 escapes cleavage by endothelial ECE-1, but this processing likely occurs by smooth muscle ECE or via alternative pathways catalyzed by chymase for ET-1 [49]. These studies suggest that ET-2 from endothelial cells is synthesized by a similar pathway. ET-3 is not released from human endothelial cells but is synthesized in other cells by ECE-1, with evidence for additional pathways [49]. ET-1 is the most abundant isoform and most studied in endothelial cells of the cardiovascular system. ET-1 is produced at lower levels by other cell types, including epithelial cells of the lungs, kidney, and colon; enteric glia cells, macrophages, and monocytes; and choroid plexus and certain neurons and reactive glial cells in the central nervous system, such as Sertoli cells, endometrial cells, hepatocytes, and breast epithelial cells [49]. The activation of ET_A_ receptors by ET-1 leads to vasoconstriction via increases in cytosolic calcium levels, whereas the activation of ET_B_ receptors through induction of endothelium-derived dilators, such as nitric oxide lead vasodilation (Figure 2). Therefore, the overall effects of ET-1 are represented by a balance between its interaction with ET_A_ and ET_B_ receptors. The hypotensive effects observed in the presence of the combined ET_A_ and ET_B_ receptor agonist suggest that the overall physiological effect of ET-1 is to increase blood pressure and maintain vascular homeostasis [50,51,52]. Figure 1, shows that the synthesis of ET-1, ET-2, and ET-3 occurs from separate mRNAs encoded by ET-1 gene variants *EDN-1*, *EDN-2*, and *EDN-3*. These preproendothelin mRNAs 1–3 are translated to form preproendothelins 1–3.

## 4. ET-1 System and AD in Minority Populations

It is well known that AD patients have reduced cerebral blood flow that precedes the associated dementia and may contribute to its progression. ET-1 has been implicated as having a role in perfusion of all organ systems in humans. ET-1 synthesis occurs in all vessels of the body and is thought to contribute to the maintenance of basal vascular tone. However, dysregulation of the ET-1 system has been implicated in the pathogenesis of inflammation, arterial hypertension, atherosclerosis, cardiovascular disease, renal failure, coronary artery disease, cerebrovascular disease, pulmonary arterial hypertension, and sepsis [50]. Male gender, African ethnicity, and older age has been associated with elevated plasma levels of ET-1.

Evans et al. observed racial and gender differences in ET-1 plasma levels. The study, which involved healthy nonsmoking AA and NHW female and male volunteers between the ages of 18 and 70, was designed to determine plasma ET-1 levels by radioimmunoassay in healthy adults. Levels of plasma ET-1 ranged from 1.8 pg/mL to 16 pg/mL (mean 6.7 pg/mL) in AAs and between 0.5 pg/mL and 12 pg/mL (mean 5.5 pg/mL) in NHWs. In addition, plasma ET-1 levels were found to be higher in women than in men (7.1 +/− 3.2 vs. 4.5 +/− 3.6; *p* < 0.01). AA men have significantly higher ET-1 levels when compared with NHW men (mean 6.2 +/− 0.8 vs. 3.7 +/− 0.4; *p* < 0.01). The authors explain that the 2-fold increase in plasma ET-1 levels could predispose AA men to left ventricular hypertrophy and other cardiovascular diseases [54]. In this study, AA women, NHW women, and AA men have similar ET-1 levels, suggesting that the higher ET-1 levels observed in women as a group would be offset by estrogen in premenopausal women that would offer protection against the detrimental effects of high ET-1 levels [54]. Grubbs et al. examined the effects of race on mRNA and protein levels of ECE-1 subisoforms, ET-1, and ET receptor profiles using saphenous vein specimens from AA (*n* = 13) and NHW (*n* = 15) patients undergoing coronary artery grafting surgery [55]. The research team concluded ECE-1a was upregulated approximately 2- and 3-fold, respectively, in AAs. ET_A_ receptor expression was higher in NHWs compared with AAs, and ET_B_ mRNA levels were 3-fold higher in AAs, suggesting that the vasoconstriction-promoting ET_B_ receptors are increased in AAs [55]. ET-1 and ECE-1 levels also were found to be higher in AAs compared with NHWs [55]. The small number of patients enrolled was a limitation in this study.

The role of the ET-1 system in AD development, particularly in minority populations, is largely unknown and represents an important knowledge gap in our understanding of the interactions between the ET-1 system and Aβ proteins in the development of AD. ET-1, a potent vasoconstrictive peptide, has been proposed as having a role in racial differences in stress reactivity [56]. In a study by Treiber et al., AAs exhibited higher absolute plasma ET-1 levels and greater increases in ET-1 in response to stressors, supporting a racial difference in hemodynamic reactivity to stress [56]. A study by Nortley et al. suggests that ET-1, which interacts with ET_A_ receptors, may cause pericyte contractions, subsequent capillary constriction, and chronic hypoperfusion of the brain microvasculature in AD patients [57]. In a study that explored whether ethnic differences in ET-1 vascular activity exist in AA and NHW adults, Campia et al. found that hypertensive AAs had enhanced ET_A_-dependent vasoconstrictor tone, likely due to increased production of ET-1 [58]. However, knowing the adverse vascular effects of sustained high levels of ET-1, this condition could predispose AAs to cerebrovascular disease that could impact development and/or progression of AD [58] (Figure 2). To our knowledge, studies involved in the dysregulation of the ET-1 system in AAs with AD largely have been unexplored. Identifying differences in ET-1 regulation in AD that induce capillary constriction by brain pericytes in AAs and NHWs would provide information to help explain racial and ethnic disparities in AD, which could contribute to the development of novel treatment strategies for targeting the ET-1 system in AAs and NHWs. The interactions between brain pericytes and the ET-1 system in AAs also deserves further investigation. We are aware that AD is a multifactorial disease and that other non-biological social risk factors that predispose AAs to AD exist, such as access to health care, poverty, being less likely to seek medical care, and clinically presenting later than ideal in the disease course [59,60,61,62]. In addition, psychosocial risk factors are thought to have a role in elevating plasma ET-1 levels that have been associated with an increased risk for cardiovascular disease [63]. In a small study, authors observed a positive correlation between chronic mental stress and elevated plasma ET-1 levels in AA males [63]. Cooper et al. observed a correlation between increased levels of plasma ET-1 in AAs with higher levels of perceived ethnic discrimination when compared with NHWs [64]. Plasma ET-1 also was shown to be higher among NHWs with low socioeconomic status [64].

## 5. ET-1 and Aβ Interactions

ET-1, a potent vasoconstrictor that likely contributes to reduced blood flow in AD ischemia, has been shown to increase Aβ production both in vitro and in vivo [65]. It has been reported that ET-1 exacerbated Aβ deposition, tau phosphorylation, and cognitive impairment after intracerebral injection of Aβ in rats. Palmer et al. discovered that the concentration of ET-1 was higher in AD than in controls in both the cerebral cortex and cerebral blood flow [66]. Cultivation and exposure of human brain endothelial cells exposed Aβ40 and Aβ42, resulting in the release of ET-1 with higher amounts observed after exposure to Aβ40 compared with Aβ42; this ET-1 release could be abrogated with the addition of superoxide dismutase [67]. Palmer et al. also observed ECE-1 activity and ET-1 levels elevated in leptomeningeal blood vessels from postmortem brains of AD patients when compared with control tissue [67]. The authors conclude that the Aβ induction of cerebral vasoconstriction likely is due in part by ECE-1 activity and ET-1 levels [67]. Nortley et al. suggest that brain vascular pericytes link Aβ to vascular dysfunction in AD [57] (Figure 2). Reports from human brain slices and a mouse model reveal that oxidative stress triggered by toxicity of Aβ causes constriction of capillaries via the generation of reactive oxygen species (ROS) by the induction of reduced nicotinamide adenine dinucleotide phosphate oxidase 4 [57]. ROS then triggers the release of ET-1, which interacts with ET_A_ receptors to cause pericyte contraction and subsequent capillary constriction [57] (Figure 2). Pericyte-mediated constriction of brain capillaries may result in chronic hypoperfusion of the brain microvasculature that likely contributes to neurodegeneration and cognitive decline observed in AD (Figure 2). An additional study by Palmer et al. suggests that the contribution of Aβ accumulation or reduction in local microvascular blood flow in AD brains by ECE-1 is a marginal finding with no significant differences in ECE-1 mRNA and protein levels in AD, vascular dementia, or control brains [68].

Paris et al. examined effects of Aβ in isolated human cerebrovessels and in a transgenic mouse model of AD to determine the effects on ET-1 levels [69]. The authors observed that enhanced ET-1 induces vasoconstriction via solubilized Aβ in isolated human middle cerebral and basilar arteries [69]. The vasoactive effects were abrogated with the cyclooxygenase-2 inhibitor SB202190, a specific p38 mitogen-activated protein kinase inhibitor (SB202190) [69]. The vasoconstrictive effects also were observed in the double transgenic APPsw AD (PS1/APPsw) mouse model that exhibited improvement upon administration of NS-398 or SB202190 [69].

## 6. Genetics and ET-1 Dysregulation and Racial Disparities in AD

The role of the gene variant *EDN-1* on AD is unknown. High plasma ET-1 levels, along with a genetic predisposition to develop vascular disease, could contribute to AD progression and pathology over a life course. A study by Liang et al. reports that a genetic link of five tagSNPs (rs6458155, rs4145451, rs9369217, rs3087459, and rs2070699) in the *EDN-1* gene to coronary artery disease is present in a Chinese Han population [70]. This is the first report of genetic polymorphisms in the *EDN-1* gene linked to an associated risk for disease in a cohort of 525 coronary artery disease patients and 675 control subjects [70]. The authors suggest that these polymorphisms are associated with high circulating levels of ET-1 [70]. Verweij et al. initiated a genome-wide association study on plasma levels of midregional proadrenomedullin and C-terminal-pro-ET-1 (CT-pro-ET-1) (a biologically stable surrogate for ET-1), which at high levels are predictors of heart disease and heart failure [71]. The team examined common genetic variants in 3444 subjects of European descent and performed genotyping for SNPs, showing levels of associations and significance for 3230 subjects; a minor variant rs2731672 was associated with higher CT-pro-ET-1 levels (*p* = 1.23 × 10^−122^ and *p* = 1.26 × 10^−67^, respectively) [71]. Furthermore, the *EDN-1* variant rs5370 was associated with elevated levels of CT-pro-ET-1 (*p* = 1.49 × 10^−27^) [71]. This study emphasizes the significance of genetic variation in the *EDN-1* variant and its precursors and their link to vascular disease. Existing genetic-linked disparities for vascular disease among minority populations in the U.S., such as hypertension, diabetes, cardiovascular disease, kidney disease, and AD, supports the notion that *EDN-1* gene variants may exist, which could result in a predisposition to having long-term high ET-1 levels, particularly in AA males. The broad implications of the long-term vasoconstrictive effects of high ET-1 levels in vascular tissues that express ET_A_ receptors likely would contribute to cerebrovascular disease that could predispose AA males to AD. Genetic polymorphisms that could lead to increased expression levels of ET-1 and its receptors (ET_A_ and ET_B_) among AA males deserves further investigation. Understanding genetic polymorphisms that lead to dysregulation of endothelins and their receptors could provide information for the development of novel therapies, as well as provide better targeting of existing therapies for minority populations who are at greater risk for AD.

## 7. Potential Therapeutics for ET-1 Dysregulation in AD

Dysregulation of ET-1 and its interactions with cognate receptors ET_A_ and ET_B_ could be important risk factors for the development and/or progression of AD. The inherent prevalence of elevated ET-1 levels in AAs could serve as a driver for racial disparities in AD. Targeting of the human endothelin system would be an important strategy for the development of novel AD treatment therapies, particularly in minority communities at greater risk. ET-1-induced vasoconstriction, resulting in cerebral hypoperfusion, may impair neuronal function, reduce Aβ and toxic metabolites clearance from the brain, and upregulate Aβ production. Endothelin receptor antagonists (ERAs) may serve a role in the attenuation or prevention of AD.

### 7.1. Endothelin Receptor Agonist and Antagonists

Studies by Palmer et al. revealed that the ERA bosentan could preserve aortic and carotid endothelial function in Tg2576 mice, suggesting that this agonist could be useful in altering low cerebral blood flow in AD [72]. Currently, three endothelin receptor antagonists are approved for use in the U.S.: (1) bosentan (2001, Tracleer^®^); (2) ambrisentan (2007, Letairis^®^); and (3) macitentan (2013, Opsumit^®^) [73]. Sitaxsentan [74], a fourth endothelin receptor blocker, was approved in Europe, but it is not approved in the U.S. and has been withdrawn from Europe due to severe liver injury linked to its use. These ERAs were developed to block the vasoconstrictive effects of endothelin-A receptors and license for the treatment of pulmonary arterial hypertension (PAH). The role of ERAs in the treatment of AD is unknown; however, both ECE-1 and ECE-2 are important for degradation and prevention of Aβ accumulation in the brains of AD patients [75,76]. Still, ECE2 null mice develop a loss of learning and memory that mimic disabilities observed in AD patients [77]. Selective antagonists (ET_A_/ET_B_) IRL-1620/PMZ-1620580 currently are being examined as novel therapies for the treatment of AD-associated pathologies [78]. Elucidation of the crystal structure of the ET_B_ receptor by Nagiri et al. revealed that the ET_B_ receptor antagonists IRL-2500 and IRL-1620.90 functioned as inverse agonists that stabilize the inactive conformation of the receptor [79,80]. This results in drug-dependent stabilization of the receptor antagonists that leads to changes in functionality, likely contributing to their effectiveness and resulting side effects. A more comprehensive review of agonists and antagonists developed over the years for the endothelin system may be found in a review by Matthias Barton and Masashi Yanagisawa [81]. Taken together, the role of ERAs in the treatment or management of AD is unknown and requires further study.

### 7.2. Endothelin Vaccines

In a study for a novel PAH treatment, Dia et al. developed a vaccine (ETRQβ-002) against the ET-1 receptor (ET_A_) ET_A_ peptide (ET_A_-002, second extracellular loop of ET_A_ receptor, conjugated to the Qβ bacteriophage virus-like particle carrier protein, ETRQβ-002) [82]. Effects of the antibodies on Ca^2+^-dependent signal transduction events were examined in monocrotaline (MCT) and Sugen/hypoxia-induced pulmonary hypertension rats [82]. Monoclonal antibodies to ET_A_-002 reduced remodeling of pulmonary arterioles and the right ventricle in MCT- and Sugen/hypoxia-induced PAH animals and decreased right ventricular systolic pressure [82]. This is a novel therapeutic approach to treat PAH, a chronic fatal disease that is associated with ET-1 dysregulation. Endothelin receptor agonist inhibits the binding of endothelin to its cognate receptor. Zhang et al. developed the monoclonal antibody getagozumab that targets the endothelin receptor ET_A_ [83]. The antibody significantly lowers pulmonary arterial pressure in both hypoxia- and MCT-induced PAH monkey models and further attenuates the pulmonary arterial and right ventricular hypertrophy in MCT-induced PAH monkeys [83]. Getagozumab was found to be safe, long-lasting, and effective in treating PAH [83].

## 8. Potential Role of Endothelin-1 and Other Endothelin-1-Related Molecules as Biomarkers in Biofluids for AD

Plasma ET-1 levels in humans have been examined extensively, however ET-1 and its converting enzymes presence in other biofluids will require further investigation. Non-invasive means of examining ET-1 and its related converting enzymes and their potential correlations with AD plasma levels of ET-1 could provide surrogate biomarkers for plasma ET-1 in AD. ET-1 is found in urine however the synthesis of urinary ET-1 is primarily made by cells of the kidney and would not correlate with ET-1 levels in plasma [84]. However, Gurusankar et al., have shown a positive correlation between saliva and plasma levels of endothelin isoforms. [85]. This study showed a statistically significant positive correlation among endothelin isoforms in saliva and plasma for big endothelin-1, ET-1, and ET-3 [85]. However, ET-2 was not significantly correlated with other isoforms in either plasma or saliva [85]. The authors suggest that there is coordinated regulation of ET-1 and ET-3 but not ET-2. Saliva ET-1 levels could serve as a non-invasive surrogate marker for plasma ET-1 levels examined in AD. The presence of ET-1 has been observed in human breast milk; however, the correlation with ET-1 isoforms in plasma requires further investigation [86]. The highest concentrations of ET-1 were observed on the third day of lactation in postpartum lactating women [87]. Foremilk samples on third day postpartum contained significantly higher concentrations of ET-1, compared to hindmilk samples [87]. However, the correlation with plasma ET-1 levels relevant to AD has not been performed.

## 9. Conclusions

Dysregulation of the ET-1 system disproportionately may affect the development of cerebral vascular disease in the brains of different ethnic populations with AD. Understanding this system could provide information to explain racial and ethnic disparities in AD, lead to the development of tools for early detection of minor cognitive disorders, and support early interventions targeting components of the ET-1 system to limit progression of AD and ADRDs. The role of endothelin receptor agonists, antagonists, and vaccines as therapeutic interventions for treatment of AD and ADRDs will require further investigation in the form of additional animal studies that could lead to randomized-controlled trials.

## Abbreviations

AAs	African Americans
Aβ	Amyloid beta protein
AD	Alzheimer’s disease
ADRD	Alzheimer’s disease and related dementias
APPsw AD	Transgenic mouse mode for Alzheimer’s disease
CT-pro-ET-1	C-terminal-pro-ET-1
DM	Diabetes mellitus
ECE-1	Endothelin-converting enzyme-1
ECE-2	Endothelin-converting enzyme-2
*EDN-1*	Endothelin-1 gene locus
*EDN-2*	Endothelin-2 gene Locus
*EDN-3*	Endothelin-3 gene Locus
ERAs	Endothelin receptor antagonists
ET_A_	Endothelin receptor type A
ET_B_	Endothelin receptor type B
ET-1	Endothelin-1
ET-2	Endothelin-2
ET-3	Endothelin-3
H/L	Hispanic/Latino
NHWs	Non-Hispanic Whites
NS-398	COX-2 inhibitor
PS1/APPsw	Transgenic mouse mode for Alzheimer’s disease
ROS	Reactive oxygen species
SB202190	Specific inhibitor of p38 MAPK signaling pathway
SCUBE2	Signal peptide-CUB-EGF-like-containing protein 2
tagSNPs	Tagged single nucleotide polymorphisms
T2DM	Type 2 diabetes mellitus
